# Investigation of miltefosine-model membranes interactions at the molecular level for two different PS levels modeling cancer cells

**DOI:** 10.1007/s10863-024-10025-y

**Published:** 2024-06-04

**Authors:** Züleyha Özçelik Çetinel, Duygu Bilge

**Affiliations:** Department of Physics, Faculty of Science, Ege University, Bornova, Izmir, 35100 Turkey

**Keywords:** Fourier transformation infrared spectroscopy, Differential scanning calorimetry, Miltefosine, Dipalmitoyl phosphatidylserine level, Cancer

## Abstract

Miltefosine (MLT) is a broad-spectrum drug included in the alkylphospholipids (APL) used against leishmania and various types of cancer. The most crucial feature of APLs is that they are thought to only kill cancerous cells without harming normal cells. However, the molecular mechanism of action of APLs is not completely understood. The increase in the phosphatidylserine (PS) ratio is a marker showing the stage of cancer and even metastasis. The goal of this research was to investigate the molecular effects of miltefosine at the molecular level in different PS ratios. The effects of MLT on membrane phase transition, membrane orders, and dynamics were studied using DPPC/DPPS (3:1) and DPPC/DPPS (1:1) multilayer (MLV) vesicles mimicking DPPS ratio variation, Differential Scanning Calorimetry (DSC), and Fourier Transform Infrared spectroscopy (FTIR). Our findings indicate that miltefosine is evidence at the molecular level that it is directed towards the tumor cell and that the drug’s effect increases with the increase of anionic lipids in the membrane depending on the stage of cancer.

**Supplementary Information:**

The online version contains supplementary material available at 10.1007/s10863-024-10025-y.

## Introduction

Disease and infection rates are steadily growing as the world’s population grows. Cancer has become a major cause of mortality globally among the different hazards to humanity (Ferlay et al. 2010; Pugazhendhi et al. 2018). 90% of cancer fatalities are caused by metastasis (Veiseh et al. 2011), and it is expected that 13 million people would die from cancer worldwide by 2030 (Pugazhendhi et al. 2018; Vyas et al. 2012). Traditional radiological and histological tests can be used to treat cancer, although chemotherapy, radiation, and surgery are the most often used cancer therapies. Treatment seeks to eliminate malignant cells while avoiding damaging healthy cells (Pugazhendhi et al. 2018; Singhal et al. 2010).

Miltefosine, edelfosine, and perifosine are synthetic anticancer alkylphospholipids (APLs) that are structurally similar to lipids and are expected to have effects on cellular membranes instead of DNA. APLs involve a lengthy hydrocarbon chain that is easily incorporated into lipid bilayer membranes, yet they are resistant to catabolic breakdown (Van Blitterswijk and Verheij 2008). As a result, APLs are thought to cumulate in cell membranes and disrupt the metabolism of normal lipid and signal transmission.

This activity, which generally results in apoptosis, is reported to be particularly successful in metabolically active, proliferating cells like tumor cell, but asymptomatic in healthy cells; that is, they have no effect on normal cells. APLs can show promising results in combination with traditional cancer treatments. For instance, it is known that ALPs make cancer cells more sensitive to radiotherapy both in vitro and in vivo (Van Blitterswijk and Verheij 2008; Sprong et al. 2001; Hilgard et al. 1997).

Miltefosine (MLT) was one of the first alkylphospholipids to enter clinical trials (Unger et al. 1988, 1990). This drug is a synthetic phospholipid derivative noted for its resemblance to lecithin, the main component of the cell membrane. It is the first antitumor lipid to be utilized in clinical studies, specifically topical medication for breast cancer skin metastases (Petit et al. 2019; Van Blitterswijk and Verheij 2013; Murray et al. 2015). It has also been demonstrated to be efficacious against a wide spectrum of malignant cells (Petit et al. 2019; Pachioni et al. 2013; Dorlo et al. 2012). It can also be used to treat bacterial, fungal, and parasitic illnesses (Petit et al. 2019; Pachioni et al. 2013; Sant’Anna et al. 2018; Rios-Marco et al. 2017).

Although the detailed mechanism of action is still unknown, its selective interaction with membrane functions is assumed (Petit et al. 2019; Verweij et al. 1992; Mollinedo 2007). In our previous study on binary systems (dipalmitoyl phosphatidylcholine/miltefosince (DPPC/MLT) and dipalmitoyl phosphatidylserine/miltefosine (DPPS/MLT) modeled on normal and cancer cells, the thermodynamic and spectroscopic effects of this drug at the molecular level were investigated. The results showed that MLT affects DPPS/MLT systems modeled cancer cells more than the DPPC/MLT system modeled healthy cells. This study showed at the molecular level that the drug tends to go after cancer cells, which means that it might have selective antitumor properties (Ozcelik Cetinel and Bilge 2022).

The investigation of the impact of such drugs on the biophysical properties of membranes is an important research field in this context.

The knowledge gained from such studies can play an essential role in designing novel drugs and delivery systems and identifying new membrane targets that could be the basis of membrane-lipid The knowledge acquired from these investigations can be used to create novel drugs and delivery systems, as well as uncover new membrane targets that might form the basis of membrane-lipid treatment (Alves et al.,2016). Due to the complexity of the cell membrane in these investigations, several mimetic model systems, and biophysical approaches have been created and applied in the research of antineoplastic drug-membrane interactions. It is vital to underline that simplifying membranes is crucial for comprehending molecular interactions and obtaining total control of such complex systems (Phillips 1972; Jones and Chapman 1995; Merz and Roux 1996).

Normal (healty) membranes feature an exterior layer made up of sphingomyalin (SM) and zwitterionic phosphatidylcholine (PC) and an interior layer made up of anionic phosphatidylserine (PS) and phosphatidylethanolamine (PE) (Yamaji-Hasegawa and Tsujimoto 2006). This asymmetric distribution, on the other hand, has not been reported for numerous cancer types (Ran et al. 2002; Ran and Thorpe 2002). Differently, it has been shown that negatively charged PS is found in the exterior layer of tumor cell membranes (Alves et al.,2016 ; Riedl et al. 2011; Zwaal et al. 2005). In the study by Riedl et al., it was revealed that the negatively charged phospholipid PS is found not only in the outer leaflets of tumor cell membranes but also in their metastases (2011). Sharma et al. discovered that an increase in blood PS exosome levels is a particular sign of cancer and that this increase is a biomarker for early-stage cancers (2017). In the literature, dipalmitoyl phosphatidylcholine (DPPC) liposomes are commonly used to mimic a healthy cell, whereas dipalmitoyl phosphatidylserine (DPPS) liposomes are utilized to mimic a malignant cell. (Souzaa et al. 2017; Riedl et al. 2017; Salisa et al. 2017; Wodlej et al. 2019; Pires et al. 2020). In addition to phosphatidylserine (PS) lipids, phosphatidylcholine (PC) lipids are also present in both cancerous and healthy cell membranes (Alves et al. 2016). Also, it is known that the amount of PS in the membrane changes depending on the stage of the malignancy (Sharma et al. 2017).

For these reasons, DPPC/DPPS (3:1) and DPPC/DPPS (1:1) lipid systems have been developed to investigate the effects of MLT on the cancer cell membrane in cases of cancer stage and/or metastasis. Thermodynamically and spectroscopically, the effects occurring at the molecular level were analyzed in depth.

## Material and method

### Chemicals

MLT, DPPC, DPPS and phosphate buffered saline (PBS) tablets were purchased from Sigma (St. Louis, MO, USA). All chemicals were obtained from commercial sources at the highest grade of purity available.

### FTIR study

MLVs were created using the method described by Severcan et al. (2000). For the DPPC/DPPS(3:1) and DPPC/DPPS(1:1) systems, appropriate amounts of DPPC and DPPS lipids were placed in round-bottomed flasks. A dried lipid film was obtained by evaporating it with a nitrogen flux and then prepare miltefosine containing liposomes, appropriate amount of miltefosine was taken from the stock solution, in which miltefosine was dissolved in ethanol, and put in a round-bottomed flask. The excess ethanol was evaporated by nitrogen stream and then pumping it for at 3 h under vacuum by using Christ LT-105 spin vac. 25 μl of PBS was supplied to round-bottomed flask with thin lipid films for FTIR measurements. MLVs were formed by vortexing the mixture at a temperature above the gel-to-fluid phase transition for 20 min. Sample suspensions of 20 μl were placed between CaF_2_ windows. Infrared spectra were collected utilizing FTIR spectrometer (PerkinElmer Frontier). 2 cm^− 1^ resolution was used with an average of 50 scans per measurement. To control the temperature, a digital temperature controller (Specac) was utilized. Samples were incubated for 5 min at each temperature before being scanned between 25 and 65^0^ C. The spectra were evaluated using the PerkinElmer v10.3.7 software. Using the PerkinElmer Spectrum One application, which is also used for data processing, the background spectrum was automatically eliminated from the spectra of the samples. The buffer spectra at various temperatures were removed from the liposome spectra at those temperatures. Using the PerkinElmer software, the subtraction procedure was carried out until the region containing the water area of roughly 2100 cm^-1^ became flat. The bands’ positions were determined by referencing the center of gravity. Bandwidth was calculated at 0.80 × peak height position. The extracted natural spectra were subjected to in-depth analysis. Experiments were repeated five times for each concentration.

### DSC study

For the DSC measurements, DPPC/DPPS(3:1) and DPPC/DPPS(1:1) MLVs were prepared in the absence and the presence of increasing concentrations of miltefosine. Thin films were obtained by hydrating appropriate amounts of DPPC and DPPS lipids (DPPC/DPPS(3:1) and DPPC/DPPS(1:1) with 50 μl PBS, pH 7.4, and the procedure mentioned above was followed. TA Q 2000 DSC device was used in DSC studies (New Castle, Delaware, USA, TA Instruments Inc.). 50 μl of the prepared samples were taken and placed in a standard aluminum DSC pan. The operating temperature range for DSC measurements for both DPPC/DPPS (3:1)/MLT and DPPC/DPPS (1:1)/MLT systems is 25–65 °C, with a 1 °C/min rate of heating. DSC analyses were performed using TA Universal Analysis 2000 software (Sariisik et al. 2019; Ergun et al. 2014; Sade et al. 2010; Sahin et al. 2013).

Calorimetric enthalpies ∆H (kJ/mol) were obtained by calculating the area under the major phase transitions. Entropy change accompanying phase transition was calculated from the transition enthalpies assuming a first order phase transition using the following equation:

$${\rm{\Delta S = \Delta H/}}{{\rm{T}}_{\rm{m}}}$$

where T_m_ is the main phase transition temperature, and ΔH is the corresponding change in enthalpy due to phase transition. Experiments were repeated five times for each concentration.

## Results

### DSC results

Calorimetric study containing miltefosine was utilized to determine the thermotropic phase behavior of the membrane and the temperature of the main phase transition of the systems, as well as the thermal properties on the phase transition curve of the membrane system. The variations in the enthalpy values and main phase transition temperature were analyzed using this data.

In the deconvolution analysis of binary systems (DPPC/MLT and DPPS/MLT), no peak was observed near to the phase transition temperature of pureDPPC MLV at concentration values greater than 24 mol% for the MLV system of DPPC/MLT. For this reason, the maximum drug concentration of the DPPC/MLT system was determined to be 24 mol%. In the same way, the maximum concentration for the DPPS/MLT system was set at 9 mol% because no peaks near the temperature of the phase transition of pure DPPS lipid were observed at concentrations higher than 9 mol% (Ozcelik Cetinel and Bilge 2022). Therefore, for ternary systems (DPPC/DPPS/MLT), 3-6-9 and 15 mol % MLT concentrations were studied.

**Fig. 1:**
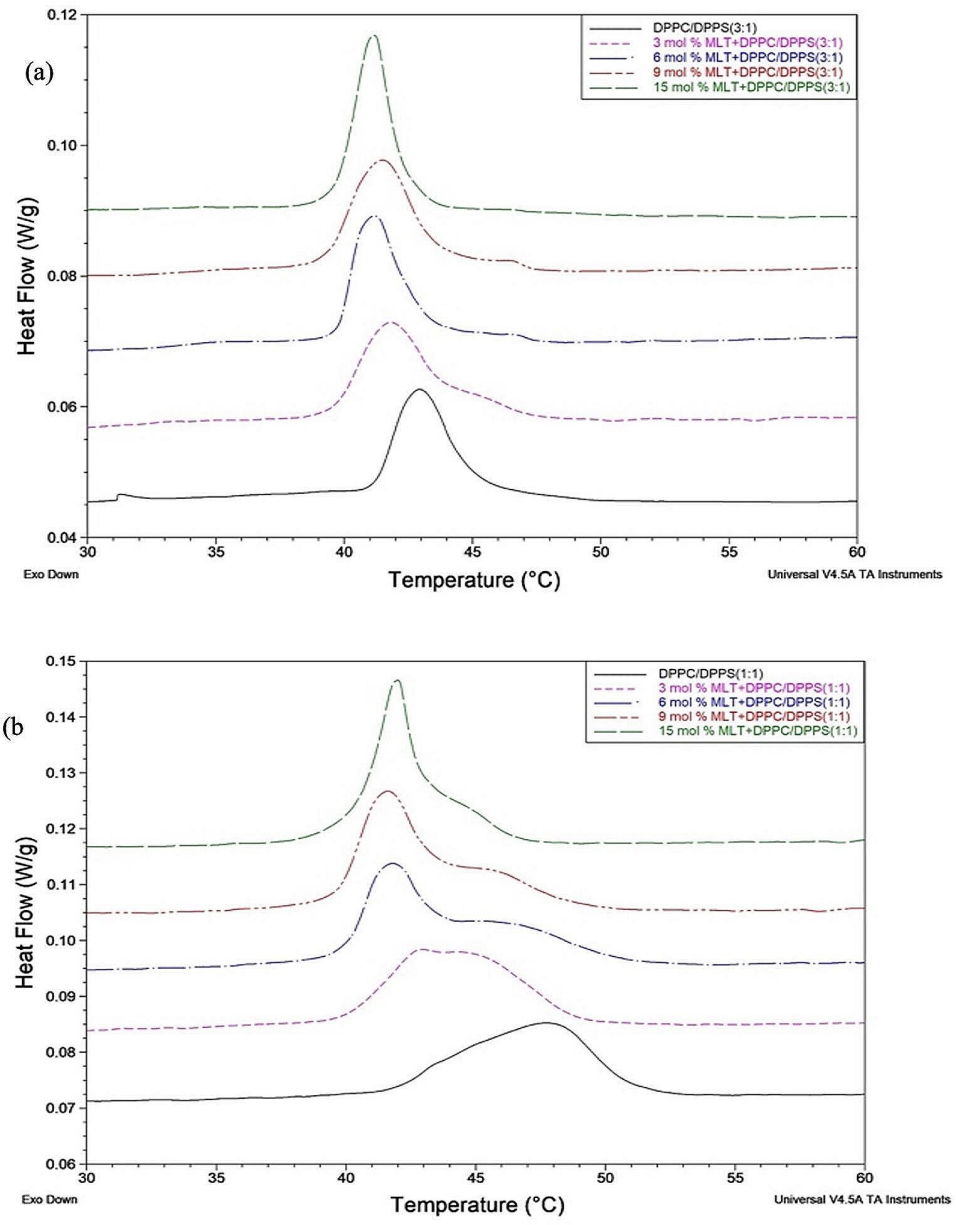
DSC thermograms of DPPC/DPPS(3:1)/MLT (**a**) and DPPC/DPPS(1:1)/MLT (**b**) systems with different mol% miltefosine

DSC thermograms of DPPC/DPPS(3:1)/MLT and DPPC/DPPS(1:1)/MLT systems are shown in Fig. 1.

Enthalpy values and the temperature of the main phase transition calculated for each peak in the DSC thermograms are given in Table 1.

**Table 1:** The main phase transition temperatures (T_m_), enthalpy (ΔH) and entropy (ΔS) values for (**a**) DPPC/DPPS (3:1) and (**b**) DPPC/DPPS (1:1) liposomes containing miltefosine

(a)	T_m_(^0^C)	∆H(kJ / mol)	∆S(kJ / mol.K)	
0 mol % MLT + DPPC/DPPS (3:1)	42.95±0.008	25.78±0.10	0.600±0.002	
3 mol % MLT + DPPC/DPPS (3:1)	41.81±0.029	37.57±0.06	0.898±0.001	
6 mol % MLT + DPPC/DPPS (3:1)	41.17±0.011	33.39±0.13	0.811±0.003	
9 mol % MLT + DPPC/DPPS (3:1)	41.49±0.025	34.14±0.09	0.823±0.001	
15 mol% MLT + DPPC/DPPS (3:1)	41.19±0.007	31.31±0.06	0.760±0.001	

(b)	T_m_(^0^C)	∆H(kJ / mol)	∆S(kJ / mol.K)	
0 mol % MLT + DPPC/DPPS (1:1)	47.43±0.02	35.29±0.12	0.744±0.003	
3 mol % MLT + DPPC/DPPS (1:1)	42.94±0.02	34.85±0.06	0.811±0.001	
6 mol % MLT + DPPC/DPPS (1:1)	41.79±0.02	34.82±0.05	0.833±0.001	
9 mol % MLT + DPPC/DPPS (1:1)	41.62±0.02	31.92±0.06	0.767±0.002	
15 mol % MLT + DPPC/DPPS (1:1)	42.00±0.06	31.45±0.01	0.749±0.001	

According to the obtained DSC results, when thermodynamic parameters such as enthalpy (∆H) values, entropy(ΔS) and main phase transition temperature (T_m_) for DPPC/DPPS(3:1)/MLT ternary systems are analyzed, with addition of MLT to the system, the T_m_ value is slightly decreased (∼ 1.78^0^C) at all MLT concentrations, and the ∆H values increase. In the thermodynamic calculations made for the DPPC/DPPS(1:1)/MLT triple system, it is seen that the T_m_ value shifts significantly to low temperatures (∼ 5.81^0^C), and the ∆H value decreases at all concentrations. Entropy values increased in both DPPC/DPPS(3:1)/MLT and DPPC/DPPS(1:1)/MLT systems when compared to the entropy values of the drug-free system ( DPPC/DPPS(3:1) and DPPC/DPPS(1:1)).

### Deconvolution analyzes

**Fig. 2:**
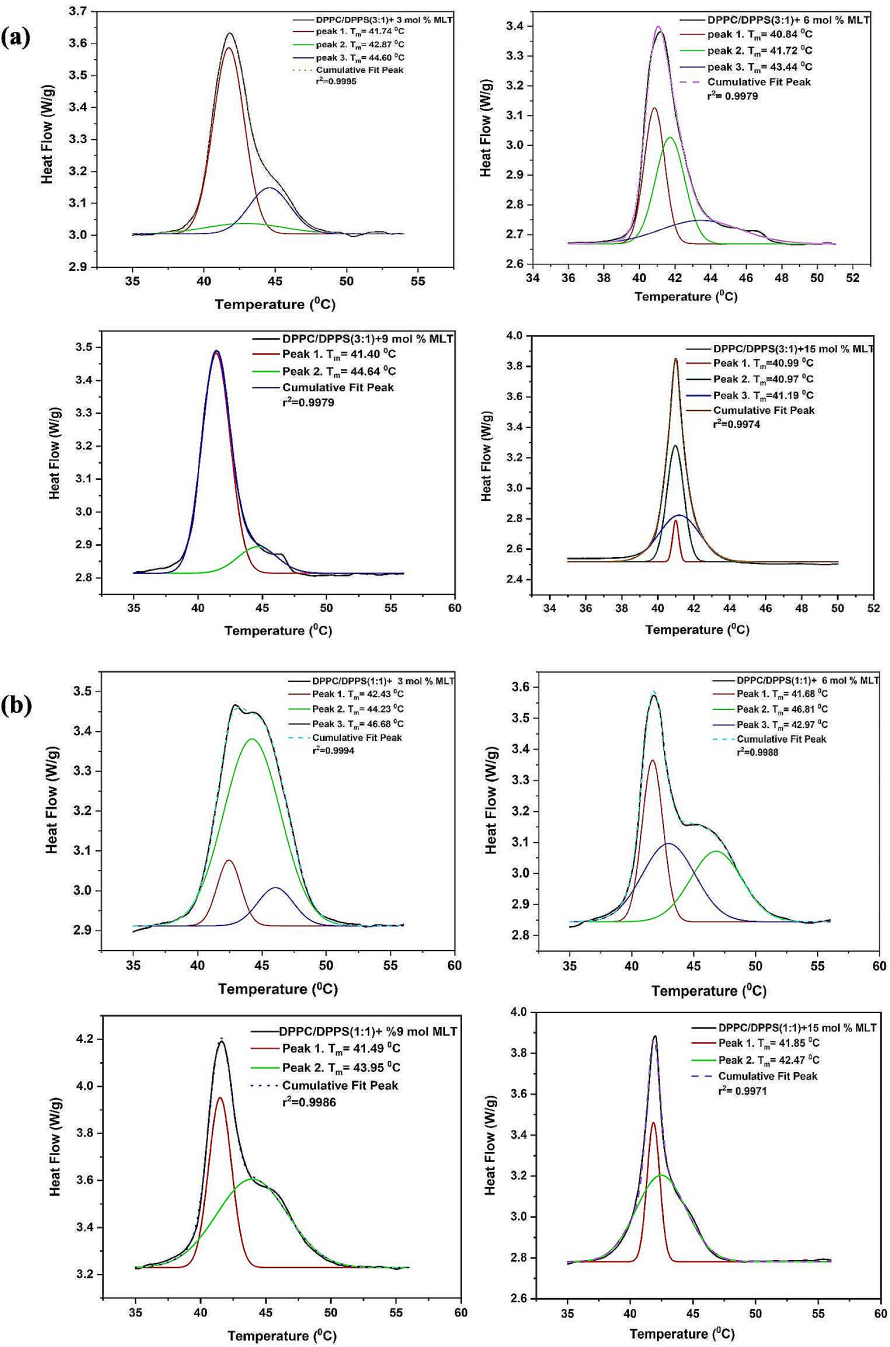
Deconvolution analyzes of (**a**) DPPC/DPPS(3:1)/MLT and (**b**) DPPC/DPPS(1:1)/MLT systems containing different mol% miltefosine

Phase separation was observed in DSC thermograms of DPPC/DPPS(3:1)/MLT and DPPC/DPPS(1:1)/MLT systems at all concentrations. Phase separation shows that there are drug-poor and drug-rich regions in the membrane. The deconvolution analyses for both systems were performed with the Origin 2018 program. Figure 2 shows the deconvolution analysis for the DPPC/DPPS(3:1)/MLT system and the DPPC/DPPS(1:1)/MLT system.

As a result of the deconvolution analysis, no peak was observed for both systems at 9 and 15 mol% MLT concentrations, close to the pure DPPC/DPPS(3:1) and DPPC/DPPS(1:1) transition temperatures of the system. The absence of a peak near the main phase transition temperature of the pure lipid system implies that these systems have reached drug saturation at the examined concentration values (Ozcelik Cetinel and Bilge 2022; Diaz and Fanani 2017). This situation shows that these concentration values are high for these systems. Therefore, in this study, it was thought that working with 3–6 mol% MLT concentration in both systems would be more appropriate.

### FTIR results

A peak near the phase transition temperature of pure DPPC/DPPS(3:1) and DPPC/DPPS(1:1) lipid mixtures at concentrations above 6 mol% was not observed in the deconvolution analysis of the DSC results of the ternary systems (DPPC/DPPS(3:1)/MLT and DPPC/DPPS(1:1)/MLT. For this reason, it was determined that the suitable maximum drug concentration for the FTIR study was 6 mol% in both systems. Accordingly, FTIR studies of ternary systems (DPPC/DPPS/MLT) were carried out with 3 and 6 mol% MLT concentrations.

Using the FTIR device’s spectra in the gel and liquid crystalline phases, the vibrational bands of the lipids’ head, interfacial region, and tail were examined. Analysis of CH_2_ antisymmetric stretching vibrational bands’ wavenumber values belonging to the tail part of lipids provides information on the membrane’s order and phase transition behavior at the molecular level. This band’s bandwidth values provide information regarding the membrane’s fluidity (dynamics) (Severcan et al. 2000, 2005; Mantsch 1984). The wavenumber values of the PO_2_^−^ antisymmetric double bond stretching band, which is related to the vibration of the lipids’ head, and the C = O stretching band, which is related to the vibrations of the lipids’ interfacial region provide knowledge about the hydration and dehydration status of the examined liposomes system at the molecular level (Lewis and McElhaney 1998; Mantsch and McElhaney 1991). The FTIR spectra of DPPC/DPPS(3:1)/MLT systems were shown in Fig S1a-b (in supplementary data), in gel (Fig. S1a) and liquid crystalline (Fig. S1b) phases.

**Fig. 3:**
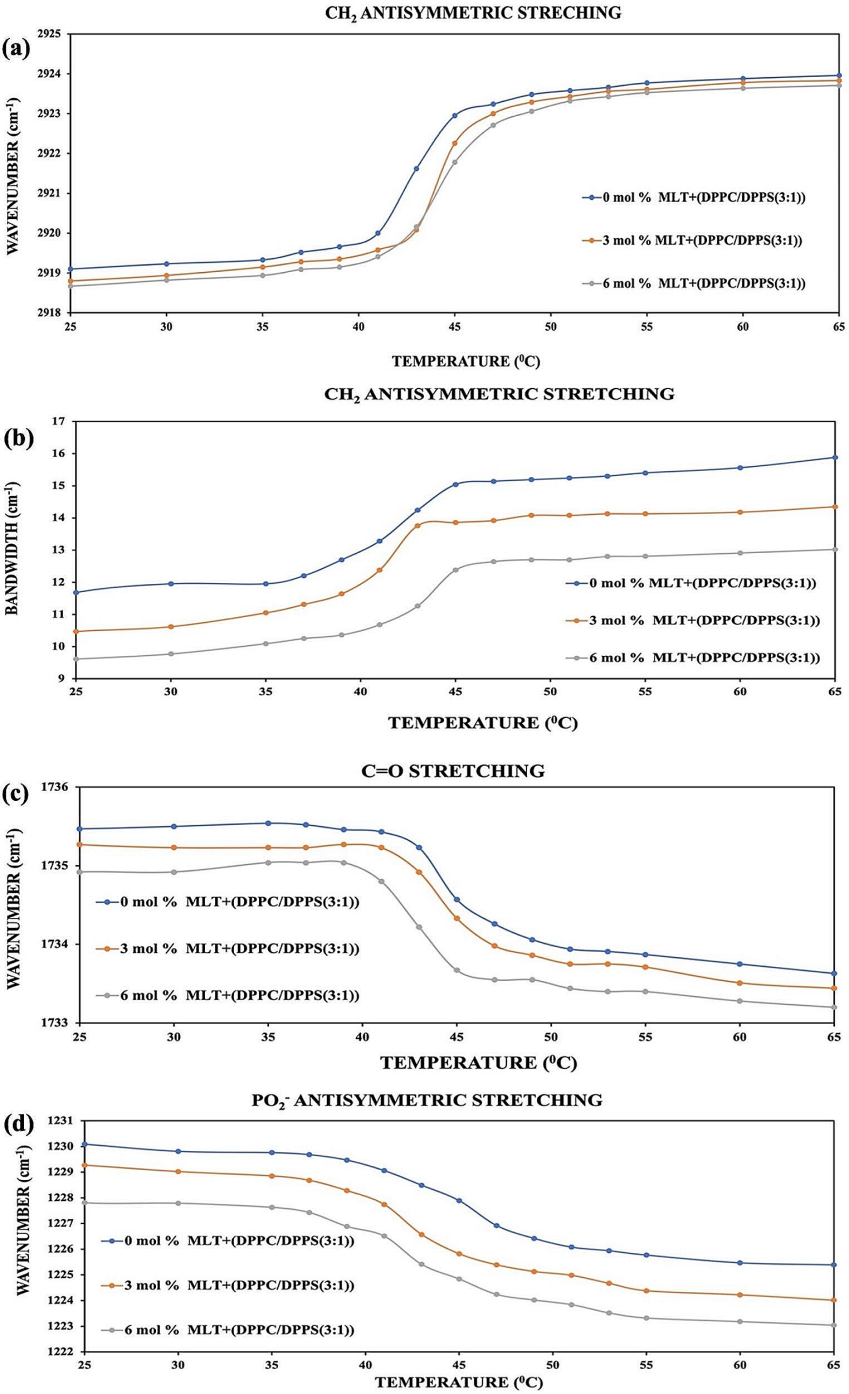
**a**, **b**, **c**, **d** illustrates the change in the vibrational bands of the DPPC/DPPS(3:1) liposomes’ head, interfacial region, and tail containing 3 and 6 mol% miltefosine concentrations

Figure 3: Temperature dependent alterations of (a) the wavenumber and (b) bandwidth values in CH_2_ antisymmetric stretching bands (c) in the C = O stretching bands (d) in PO_2_^−^ antisymmetric double bond stretching in DPPC/DPPS(3:1) MLVs with and without 3 and 6 mol% MLT.

Figure 3a depicts a considerable rise in wavenumber values for drug-free DPPC/DPPS(3:1) system at 42.95 °C, showing the transition from gel to liquid-crystalline phase. When the CH_2_ antisymmetric vibration band’s wavenumber values were examined as a function of temperature (Fig. 3a), in the gel and liquid crystal phases, the wavenumber values of the systems containing miltefosine were found to be lower than those of the systems without miltefosine. In other words, it has improved the order of the lipids.

As shown in Fig. 3b, adding MLT to pure (drug-free) systems decreases the bandwidth values of DPPC/DPPS(3:1) MLVs in gel and liquid crystalline phases at both concentrations, reducing system fluidity.

Figure 3c shows a decrease in the wavenumber values of the C = O stretching bands in both miltefosine doses at gel and liquid crystalline phases. This means that miltefosine either strengthens the hydrogen bonds already around the interface region or makes new hydrogen bonds at all MLT concentrations in both phases.

The presence of MLT in the system reduced the wavenumber in both phases as seen in Fig. 3d. These findings indicate that new hydrogen bonds are generated or that existing hydrogen bonds are strengthened in the system.

The FTIR spectra of pure DPPC/DPPS(1:1), containing 3 and 6 mol% miltefosin doses for the DPPC/DPPS/MLT system were shown in figure S2a-b, in gel (Fig. S2a) and liquid crystalline (Fig. S2b) phases.

**Fig. 4:**
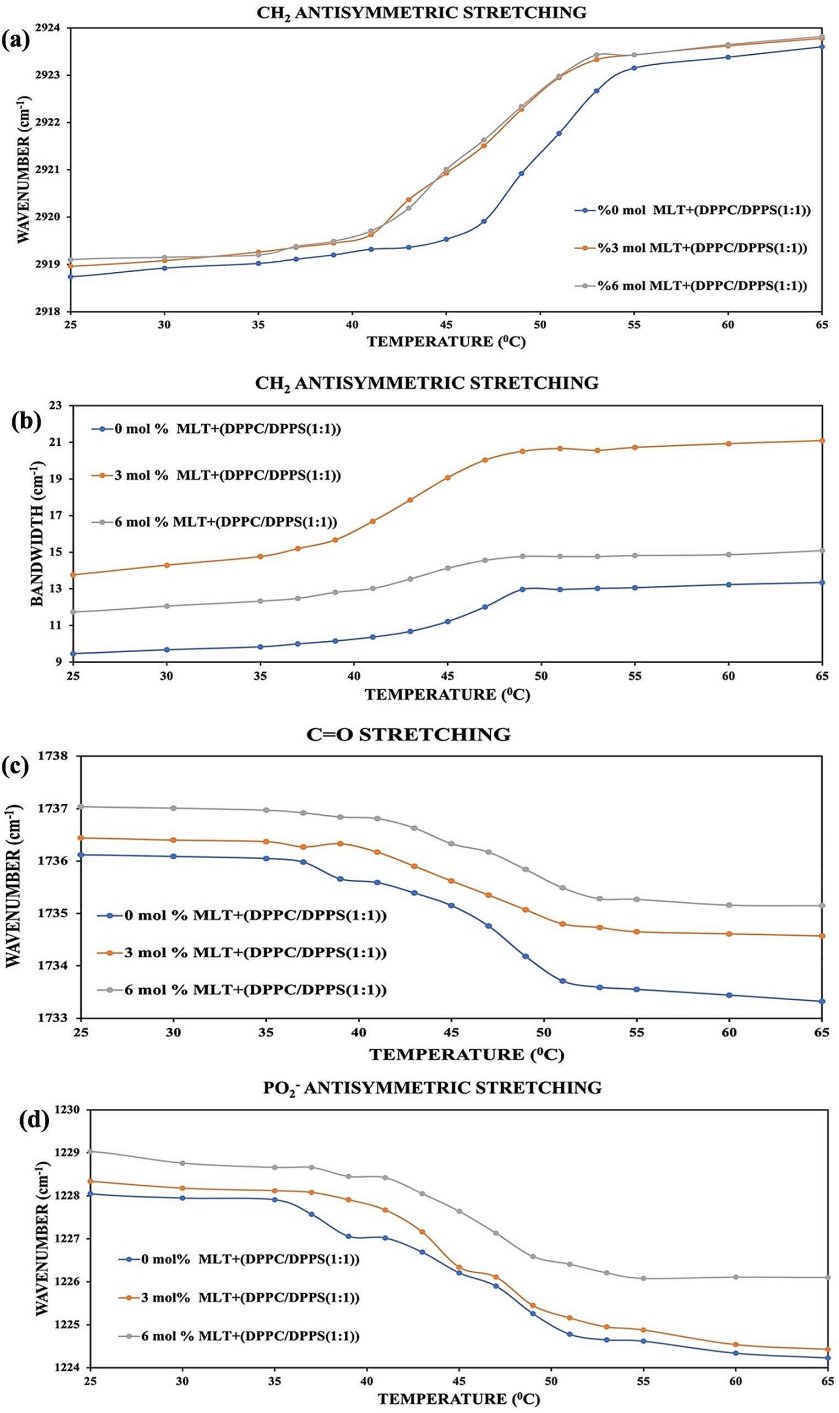
**a**, **b**, **c**, **d** shows the change in the vibrational bands of the DPPC/DPPS(1:1) liposomes’ head, interfacial region, and tail containing 3 and 6 mol% MLT concentrations

Figure 4: Temperature dependent alterations of (a) the wavenumber and (b) bandwidth values in CH_2_ antisymmetric stretching bands (c) in the C = O stretching bands (d) in PO_2_^−^ antisymmetric double bond stretching in DPPC/DPPS(1:1) MLVs with and without 3 and 6 mol% MLT.

The main phase transition temperature (T_m_) of the pure DPPC/DPPS(1:1) liposome is ∼ 47.43 °C. Miltefosine added to the system increased in the CH_2_ antisymmetric stretching band’s wavenumber values at both doses, as shown in Fig. 4a, at temperatures corresponding to the gel and liquid crystal phases, indicating that MLT decreased the membrane order in both phases.

Miltefosine increases DPPC/DPPS(1:1) MLVs’ bandwidth values at all doses, as seen in Fig. 4b, in both phases (in the gel and liquid-crystalline phases). As a result, MLT increases the dynamics of DPPC/DPPS(1:1) lipids at all doses.

All MLT doses applied to the membrane system resulted in the increased wavenumber values, as seen in Fig. 4c. This demonstrates that the hydrogen bonding capability of DPPC/DPPS(1:1)/MLT systems reduces in the glycerol backbone region (C = O stretching vibration band area), and there are free carbonyl groups surroundings; hence, dehydration happens around this functional group.

As seen in Fig. 4d, both miltefosine concentrations in the both phases increased the wavenumber. This indicates that adding miltefosine to the system reduces the hydrogen bonding capacity.

## Discussion

Liposomes are small, spherical artificial vesicles. They can be made from cholesterol and natural, non-toxic phospholipids. Liposomes are a promising system for drug delivery due to their size, hydrophobicity and hydrophilicity, as well as their biocompatibility. Liposomes are a very good model of a biological membrane and are highly suitable to assess different biophysical parameters and are by far the most used models to study anticancer drug membrane interactions. Vesicles with one or more phospholipid bilayer membranes can transport aqueous or lipidic drugs, depending on the drug type. Lipids are amphipathic, having both hydrophobic and hydrophilic properties in aqueous media, so their thermodynamic phase properties and self assembling characteristics influence entropically focused confiscation of their hydrophobic sections into spherical bilayers. These lipids have their own phase transition temperatures. This phase transition temperature differs due to the melting of the acyl chains of the phospholipids. T_m_ denotes the main phase transition temperature, which is defined as the temperature at which the gel phase transitions to the liquid crystal phase. This temperature can be measured with the DSC device. The peak in the calorimetric thermogram obtained from pure phospholipids is sharp and narrow. The sharp and narrow peak is caused by the lipid chains cooperating (fusion) in a range of the limited temperature. Interactions between the membrane’s tail and the drugs utilized may affect the membrane’s phase transition profile (Mady et al. 2012; McElhaney 1982).

The temperatures (T_m_) of the main phase transition for the drug-free DPPC/DPPS(3:1) and DPPC/DPPS(1:1) systems are 42.95 ^0^C and 47.43 ^0^C, respectively. This transition’s enthalpy values are 34.85 J/g and 47.21 J/g, respectively. When MLT was included in the pure membrane systems, the thermotropic behavior of the membrane altered. Since the change in the thermodynamic behavior of the lipid depends on the added molecule and its concentration, even a small amount of additive changes the T_m_ by changing the phase transition at a certain temperature range. It can also change the system’s fluidity or solidity (Jain and Wu 1977). This decrease in the T_m_ value implies a significant interaction between the drug molecules and the lipid’s hydrocarbon chain (Canseco and Casas 2020). T_m_ value (∼ 1.78^0^C) decreases slightly with increasing concentration in the DPPC/DPPS(3:1)/MLT system. In contrast, a significant decline in T_m_ value (∼ 5.81 ^0^C) is observed in the DPPC/DPPS(1:1)/MLT system. The main transition temperature(T_m_), where the heat capacity (Cp) reaches its maximum value, corresponds to the phase transition from gel to liquid-crystalline (Arrieta et al.2020.). The shift of the T_m_ value to lower temperatures indicates that the liquid crystal phase is preferred, that is, the membrane becomes more fluid, which is evidence that there is a strong interaction between the acyl groups of the agent and the liposome (Canseco and Casas 2020). At the same time, the broadening of the main phase transition peak indicates decreased the cooperativity (size of cooperativity unit) between lipid bilayers (Harris et al. 1995; El Maghraby et al. 2005). In the DPPC/DPPS(3:1) system, the phase transition temperature of the drug-free system is 42.95 ℃. When the MLT concentration is increased, the main phase transition temperature of each concentrated system creates a change of at most ∼ 1.78 ℃ (6 mol % MLT) compared to the main phase transition temperature of the drug-free system. In the DPPC/DPPS(1:1) system, this change is ∼ 5.81 ℃ (9 mol % MLT). This significant decrease in T_m_ is seen as significant phase separation and broadening in the main phase transition peaks in the DSC thermograms in Fig. 1b. When the two systems are compared with each other, the lowest T_m_ value for the DPPC/DPPS(3:1)/MLT system is seen at 6 mol%, while the lowest T_m_ value for the DPPC/DPPS(1:1)/MLT system is seen at 9 mol%. This situation can be associated with the increase in the number of anionic lipids in the structure with the increasing DPPS ratio in the system. The anionic charged lipid molecules in the structure repel each other strongly. At the same time, the diameter of the head groups of DPPS lipids is smaller than that of DPPC, and the distance between DPPS lipids is greater due to the anionic charge of DPPS lipid molecules. Therefore, when the DPPS ratio increases in the DPPC/DPPS/MLT system, the MLT drug enters the cooperative region of the lipids (C_2_-C_8_) more easily, increasing the lipid-drug interaction, and lower T_m_ may occur at higher concentrations.

Deformation is seen in the peaks at all MLT concentrations for DPPC/DPPS(3:1)/MLT and DPPC/DPPS(1:1)/MLT systems, as shown in Fig. 1a-b, and a secondary peak appears, which is known as phase separation. At doses where phase separation arises, it produces drug-rich and poor zones in liposomes (Severcan et al. 2005). The concentration at which phase separation is seen in the DSC curves is defined as the additional critical concentration value (ACC) at which the membrane reaches drug saturation. This situation gives the concentration at which the transition from spherical liposome structure (with a lower concentration of drug) to tubular liposome (rich in drug) begins. Spherical and tubular liposome structures exist together at the concentration value (ACC value) where there is phase separation, that is, two peaks are seen (Arrieta et al. 2020; Garidel et al. 2000). Knowing the ACC is an important step in the design of liposomal release systems, especially if another drug has to be included in the formulation, as it relates to the morphology of the liposome, the charge capacity of hydrophobic molecules, the discharge of hydrophilic molecules, and the circulation time in the bloodstream (Zhang et al. 2018).

For this reason, it is important to perform deconvolution analysis at the peaks where phase separation is observed because each sub-peak contributes to the thermodynamic parameters. When the absolute peak is divided into sub-peaks in deconvolution analysis, non-appearance of a sub-peak at a temperature near to the drug-free (pure system) lipid’s phase transition temperature indicates that the system has approached saturation with the drug (Diaz and Fanani 2017). There is no peak near to the temperature of the main phase transition of the pure systems (DPPC/DPPS(3:1) and DPPC/DPPS(1:1) at concentrations over 6 mol% MLT in both DPPC/DPPS(3:1)/MLT and DPPC/DPPS(1:1)/MLT systems in the deconvolution analyses. This showed that working with 3–6 mol% MLT concentration would be more appropriate for both systems.

With increasing drug concentration, the DPPC/DPPS(3:1)/MLT systems appear to have a higher enthalpy and entropy than the pure liposome. In other words, by adding miltefosine to the system, the mobility of phospholipid chains is restricted and the the hydrophobic interaction between lipids has increased. Breaking down such a structure requires more energy (Arrieta et al. 2020). The increase in ΔH may be due to the partial interdigitation of MLT molecules in the membrane. As reported in the literature, the main cause of interdigitation is the increased lateral area between lipid headgroups with the formation of cavities in the hydrophobic core. Through interdigitation the system gains energy due to the stronger van der Waals interactions in the interior of the membrane bilayers and due to an entropy gain by replacing the highly ordered water molecules at the interface with the polar part of the amphiphilic molecules (Chiou et al. 1992; Kranenburg et al. 2004). This may be an indication of strong lipid-lipid interaction in the tails of the DPPC/DPPS(3:1) system. Such an effect organizes the system and explains the high amount of energy required to transition into liquid crystalline states, which is reflected by the increased enthalpy. In such a case, the amino group (pKa = 10.17) is expected to be protonated as a result of the drug interacting with the polar head groups of the membrane (Konstantinidi et al. 2018). For the DPPC/DPPS(1:1)/MLT systems, the opposite is true; increasing miltefosine concentrations cause a significant decrease in enthalpy. For the DPPC/DPPS(1:1)/MLT systems, the converse is true; increasing miltefosine concentrations cause a significant decrease in enthalpy. This decrease in enthalpy values (ΔH) shows that lipid-drug interactions are increasing and van der Waals interactions between phospholipid are decreasing. Entropy values increase for DPPC/DPPS(1:1)/MLT system. Consequently, the disorder of the systems may increase (Ali et al. 2000; Maswadeh et al. 2002). As a result of an increase in the DPPS ratio in the structure, an increase in the number of anionic lipids occurs. The repulsion of negatively charged head groups causes an increase in the distance between lipid molecules. Therefore, the miltefosine molecule may penetrate the lipid interaction region more easily (C_2_-C_8_). As a result, MLT interacts more in DPPC/DPPS(1:1) systems than in DPPC/DPPS(3:1) systems.

With FTIR spectroscopy, crucial structural information, such as the conformational order, fluidity (dynamics) and hydration-dehydration effects on the head groups and backbones of the membranes, can be obtained. The conformational order and phase transition behavior of the liposome are revealed by the wavenumber variation of the examined CH_2_ antisymmetric stretching vibrations. The wavenumber values of this band are dependent on the order of phospholipid’s acyl chains and are utilized to determine the average trans and gauche isomerization of the membrane. All trans conformers in the membrane system become gauche confermers as the membrane transitions from gel to liquid crystal phase (Umemura et al. 1980; Casal et al. 1984). In DPPC/DPPS(3:1)/MLT systems, it was observed that the wavenumber values of CH_2_ antisymmetric stretching vibrations at both doses decreased when compared to the wavenumber values of the drug-free system. This indicates that MLT increases the order of membranes. This is due to the increased interactions between phospholipids in the liposome.

Because of the increased contacts between phospholipids in the membrane, phospholipid chain motions are limited, and so hydrophobic interactions between lipids increase. The situation is the opposite for DPPC/DPPS(1:1)/MLT systems. MLT added to the system increased the wavenumber values of CH_2_ antisymmetric stretching vibrations bands in both phases. In other words, MLT added to membranes disrupts the order of the DPPC/DPPS(1:1)/MLT systems. The increase in system disorder demonstrates that lipid-drug interactions increase but van der Waals interactions between phospholipid are decrease (Ali et al. 2000; Maswadeh et al. 2002). The shift in the bandwidth values in the CH_2_ antisymmetric stretching band area in the FTIR spectrum provides knowledge on the dynamics and fluidity of the systems consisting of membranes (Severcan et al. 2000; Casal and Mantsch 1984).

The bandwidth values of the CH_2_ antisymmetric stretching band decrease for the DPPC/DPPS(3:1)/MLT system in all phases with added MLT concentrations, while they increase for the DPPC/DPPS(1:1)/MLT system. This shows that MLT reduces the fluidity (dynamics) of DPPC/DPPS(3:1)/MLT systems while increasing the fluidity of DPPC/DPPS(1:1)/MLT systems.

When the samples’ PO_2_^-^ antisymmetric double bond stretching and C = O stretching bands from the DPPC/DPPS(3:1)/MLT and DPPC/DPPS(1:1)/MLT systems are analyzed, hydration is observed at all concentrations in both phases in both regions for the DPPC/DPPS(3:1)/MLT systems. In contrast, dehydration is observed in both phases and at all doses for the DPPC/DPPS(1:1)/MLT systems in both areas.

Consequently, with the increase of DPPS ratio in the systems, significant degradation in DSC thermogram peaks and a significant decrease in T_m_ values (∼ 5 ^0^C) were observed. In addition, there was a decrease in the order of the systems and dehydration in the hydrophilic head (PO_2_^−^ double stretch band region) and backbone (C = O stretch region). In DPPC/DPPS (3:1) systems, the excess of DPPC molecules causes the system to be more tightly packed. Because the head diameter of DPPC lipids is larger than that of DPPS lipids, and at the same time, since they are zwitterionic, the distance between lipid molecules in the system is less. Increasing the number of anionic charged lipids in the system (as in the DPPC/DPPS(1:1) system) causes an increase in the repulsive forces between the lipids, increasing the distance between the lipid molecules. Opening the distance between the head groups of lipid molecules causes miltefosine molecules to enter the cooperation region ((C_2_-C_8_)) of the membrane more easily. According to all these results, it can be said that miltefosine increases the drug-lipid interaction by entering in the hydrocarbon region of the DPPC/DPPS(1:1) systems (the cooperativity region (C_2_-C_8_)) more easily than the DPPC/DPPS(3:1) systems.

## Conclusion

The increase in the DPPS ratio in ternary systems increases the drug-lipid interaction by causing the drug to enter the tail part of the lipids more easily. In other words, MLT affects DPPC/DPPS(1:1)/MLT more than DPPC/DPPS(3:1)/MLT ternary systems. The increase in the DPPS ratio in the structure is related to the early stage of cancer or the metastasis status. According to the results of clinical studies, it is thought that alkylphospholipids (APL) kill tumor and therefore they are very sensitive to cancerous cells. In contrast, it is thought that they do not affect healthy cells. Results from this study, which is a continuation of our research with binary systems modeling the interactions of MLT with healthy and cancerous cells separately, showed that our results are the first study at the molecular level to support that MLT has the property of directing towards tumor cells and that the interaction increases with the increase in PS ratio, which is a marker of cancer stage and metastasis. Membrane properties (either neutral or charged lipid) also modulate drug penetration, conformation, and/or location within the membrane and, therefore, affect the therapeutic target. MLT will also have different effects on the physiological properties of the membrane, including changes in lipid conformation, surface charge, lipid domains and packaging, membrane fluidity and curvature, and consequent cell function. A better understanding of the interactions of MLT with phospholipid membrane systems at the molecular level can be obtained by examining the data presented here. Therefore, it is thought that this study will contribute to the development of more advanced treatment systems.

### Electronic supplementary material

Below is the link to the electronic supplementary material.

Supplementary Material 1

## Data Availability

No datasets were generated or analysed during the current study.
