# Supplementary material for: Investigation of miltefosine-model membranes interactions at the molecular level for two different PS levels modeling cancer cells
Source: J Bioenerg Biomembr. 2024 Jun 4;56(4):461–73. doi: 10.1007/s10863-024-10025-y (PMC11217121; doi:10.1007/s10863-024-10025-y)
Supplement: Supplementary file 1 — Supplementary Material 1 [file 10863_2024_10025_MOESM1_ESM.docx]

**Supplementary Information**

**Investigation of miltefosine-model membranes interactions at the molecular level**

**for two different PS levels modeling cancer cells**

**Züleyha Özçelik Çetinel, Duygu Bilge**

*Department of Physics, Faculty of Science, Ege University, 35100 Bornova, Izmir, Turkey*

Corresponding Author: [duygu.bilge@ege.edu.tr](mailto:duygu.bilge@ege.edu.tr)


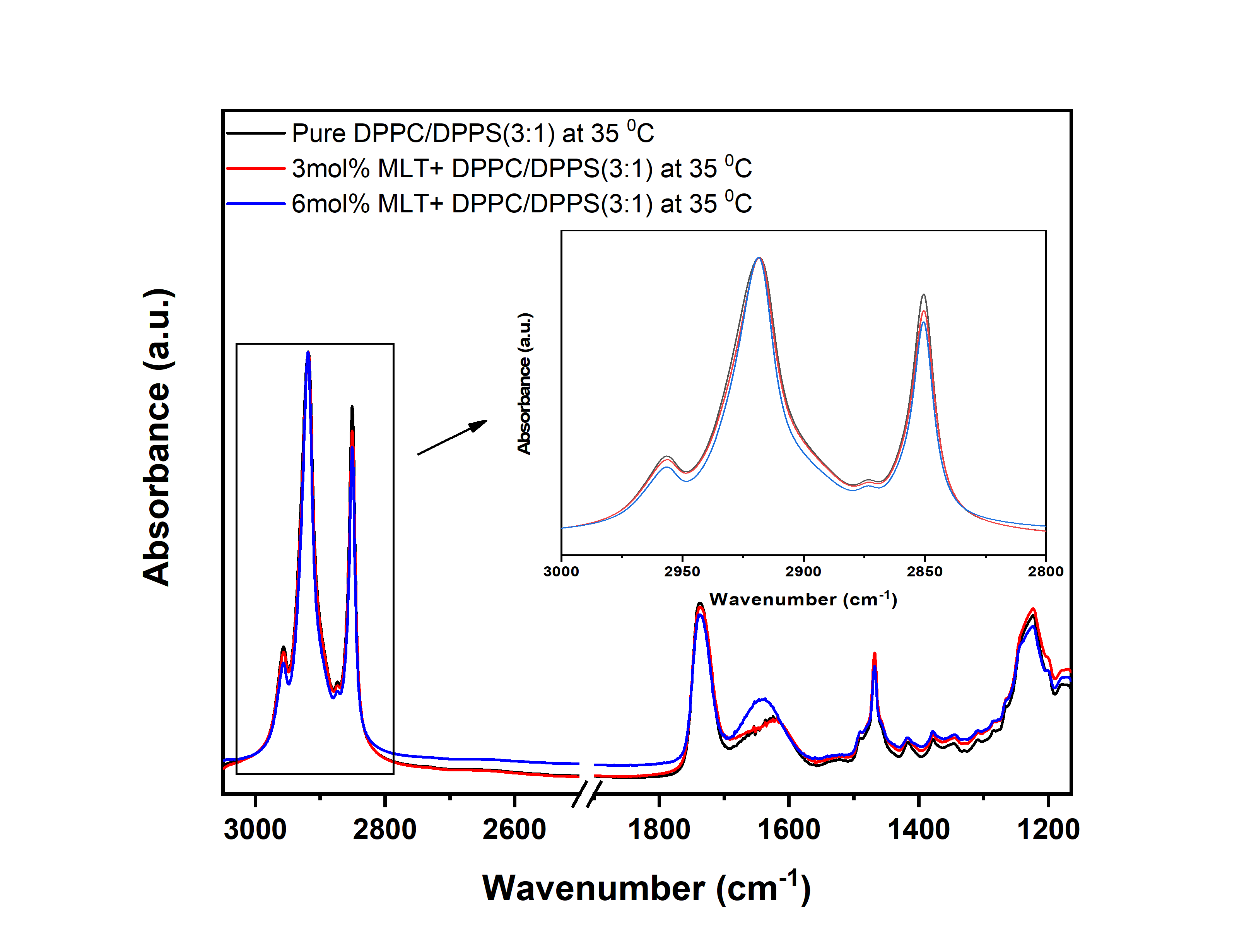

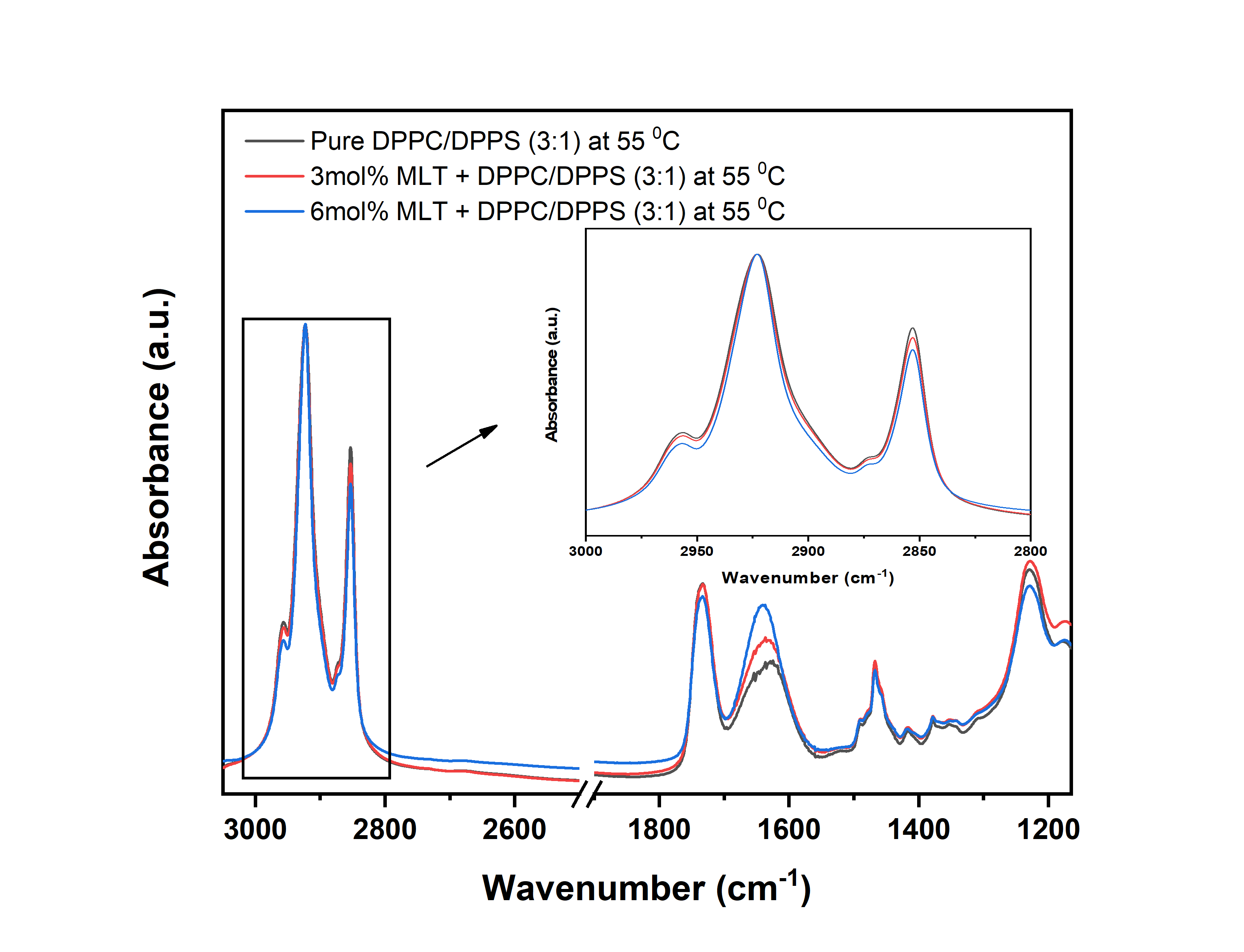


(a)

(b)

**Fig. S1.** FTIR spectrum of DPPC/DPPS(3:1) systems in (a) with and without 3 and 6 mol% MLT concentration at 35^0^C ( gel phase), (b) with and without 3 and 6 mol% MLT concentration at 55^0^C (liquid crystalline phase).


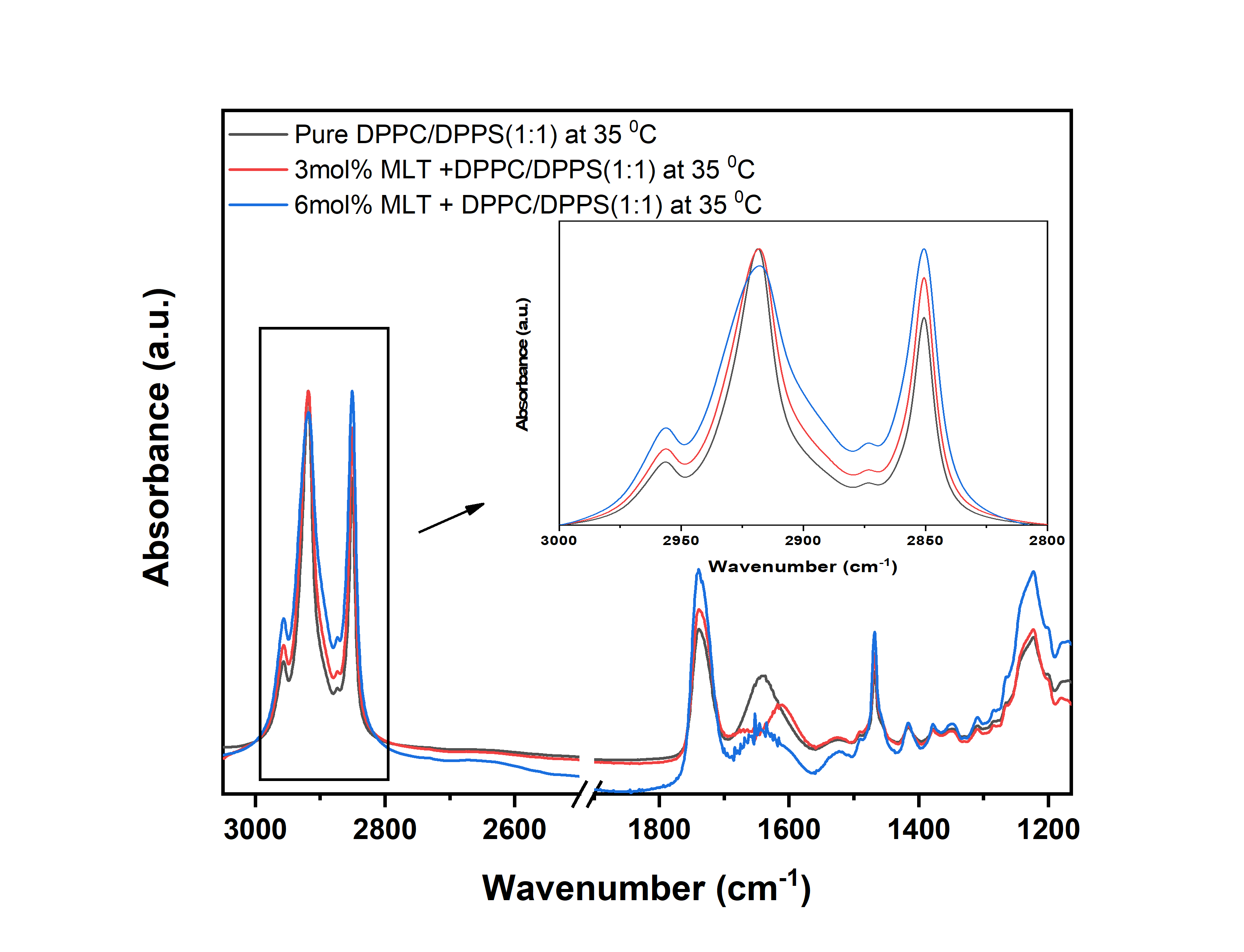

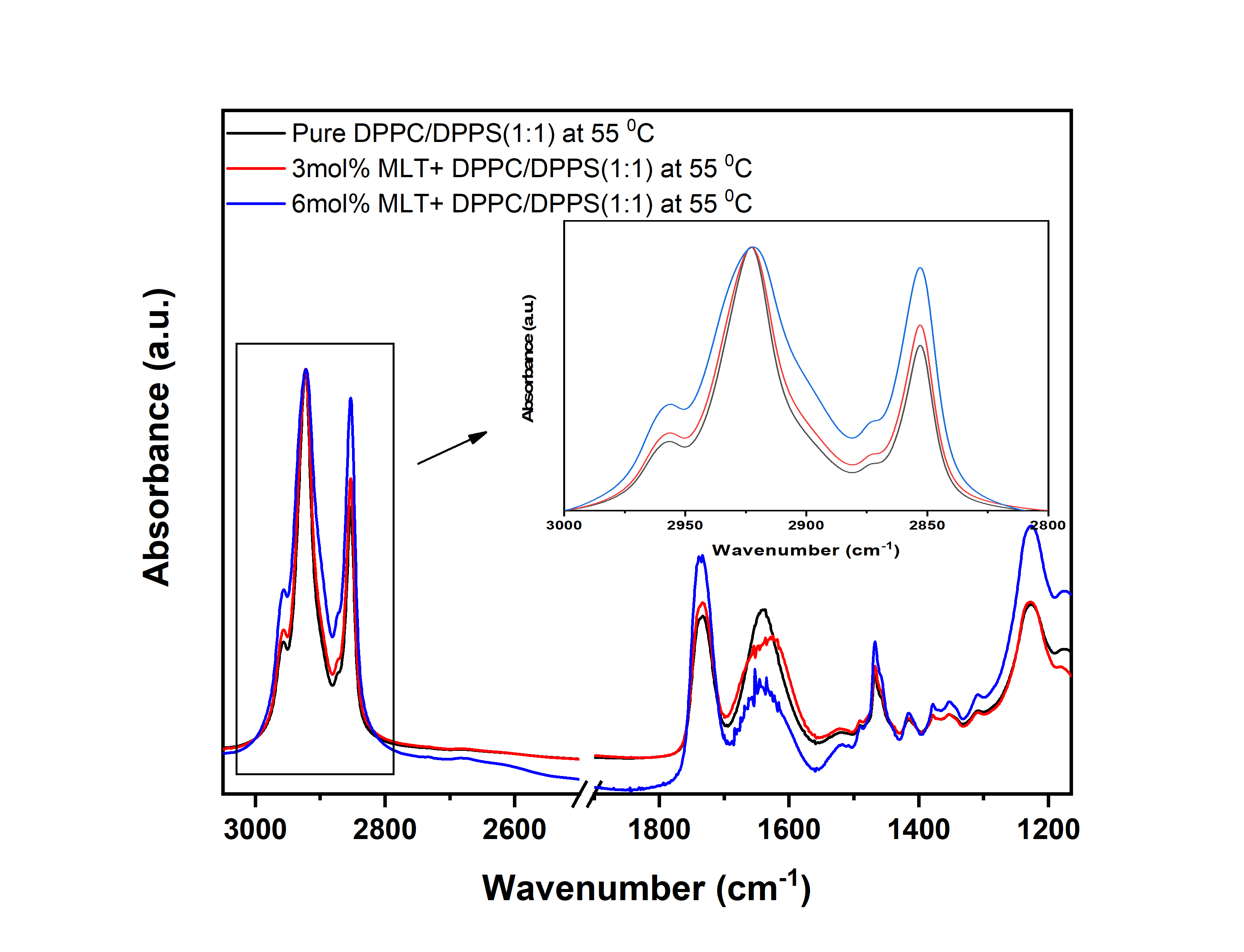


(a)

(b)

**Fig. S2.** FTIR spectrum of DPPC/DPPS(1:1) liposomes in (a) with and without 3 and 6 mol%MLT concentration at 35^0^C (gel phase), (b) with and without 3 and 6 mol% MLT concentration at 55^0^C (liquid crystalline phase).

As seen in Figure S1 and S2 , miltefosine caused a change in the wavenumber and bandwidth values of the FTIR vibration bands at the gel (35^0^C) and liquid crystalline (55^0^C) phases of the membranes. This type of change may be utilized to collect information on the system’s numerous physicochemical processes. For each sample, the vibration bands of the tail, head and glycerol backbone regions of the liposome obtained from the FTIR spectrum were analyzed in detail.


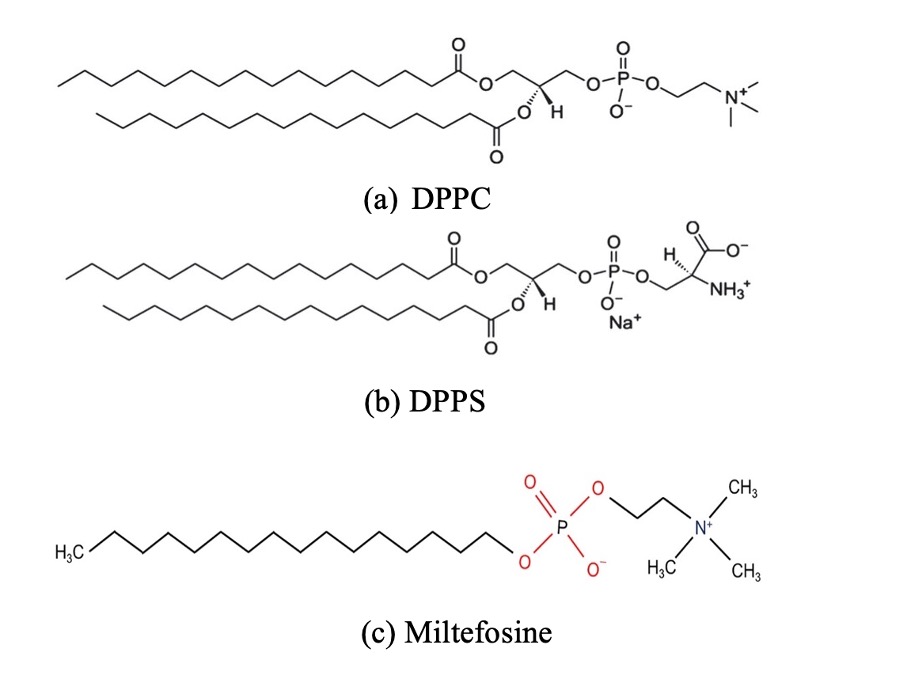


**Fig. S3.** Chemical structure of **(a)** Dipalmitoyl phosphatidylcholine (DPPC), **(b)** Dipalmitoyl phosphatidylserine (DPPS), **(c)** Miltefosine (MLT)
